# Analysis of Costs per Responder in US Adults with Paroxysmal Nocturnal Hemoglobinuria with a Suboptimal Response to Prior Eculizumab Treatment

**DOI:** 10.3390/hematolrep15040060

**Published:** 2023-10-13

**Authors:** Jesse Fishman, Seri Anderson, Sandra E. Talbird, David Dingli

**Affiliations:** 1 Apellis Pharmaceuticals, Inc., Waltham, MA 02451, USA; jesse.fishman@apellis.com; 2 RTI Health Solutions, Research Triangle Park, NC 27709, USA; stalbird@rti.org; 3 Division of Hematology, Department of Internal Medicine, Mayo Clinic, Rochester, MN 55905, USA; dingli.david@mayo.edu

**Keywords:** paroxysmal nocturnal hemoglobinuria, pegcetacoplan, eculizumab, cost analysis, response analysis

## Abstract

European Society for Blood and Marrow Transplantation (EBMT) hematologic response categories comprehensively assess complement inhibitor responses in patients with paroxysmal nocturnal hemoglobinuria (PNH). Using data from the 16-week randomized controlled period of the phase 3 PEGASUS trial (N = 80), we estimated the treatment cost per responder by the EBMT response category for pegcetacoplan and eculizumab in adults with PNH and a suboptimal response to eculizumab. Average drug costs per responder, number needed to treat, and incremental drug costs per responder were estimated using dosages administered during the trial (base case). A US payer perspective (2020 US dollars) was used. Scenario analyses were conducted for various costs, dosages, treatment durations, patient populations, and settings. In total, 30 of 41 (73%) who switched to pegcetacoplan and 2 of 39 (5%) patients who continued eculizumab had a good, major, or complete response (good-to-complete responders) at Week 16. Average weekly drug costs per good-to-complete responder were USD 15,923 with pegcetacoplan and USD 216,100 with eculizumab; average weekly drug costs per patient were USD 11,651 and USD 11,082, respectively. Average drug costs per good-to-complete responder with pegcetacoplan were similar across complement inhibitor-naïve populations and were consistently lower than with eculizumab. Switching from eculizumab to pegcetacoplan allowed more patients with a suboptimal response to attain a good-to-complete response at lower costs. These results apply to patients with a suboptimal response to prior eculizumab treatment only.

## 1. Introduction

Paroxysmal nocturnal hemoglobinuria (PNH) is a rare, chronic disease that results in complement-mediated intravascular and extravascular hemolysis, leading to fatigue, hemolytic anemia, thrombosis, and peripheral blood cytopenias [1]. Patients with PNH usually require lifelong treatment [2]. Managing PNH is complex, in part because there is a lack of formal clinical guidelines [3]. Further, before the 2007 US Food and Drug Administration (FDA) approval of eculizumab (Soliris), a complement component 5 (C5)-directed monoclonal antibody, only supportive care was available for patients with PNH [3,4,5]. Eculizumab greatly reduced PNH symptoms and mortality, becoming the standard of care [3,6].

Treatment options have increased further, with three complement inhibitors approved in the United States for PNH treatment: eculizumab, ravulizumab (Ultomiris), and pegcetacoplan (Empaveli) [7]. Eculizumab and ravulizumab are intravenous (IV) C5 inhibitors that target intravascular hemolysis [8,9]. Pegcetacoplan is a subcutaneous (SC) complement component 3 (C3)-targeted therapy that acts earlier in the complement cascade to address both intravascular and extravascular hemolysis [10,11].

With several PNH therapies available, clinicians and formulary decision-makers may need to weigh the costs and clinical outcomes of each therapy to quantify their relative value. Therapeutic decisions for patients with PNH may benefit from a careful economic evaluation because of the high expense of treating PNH with complement inhibitors, which approaches USD 500,000 per patient each year [12,13].

Without complement inhibitor therapy, patients with PNH experience pronounced symptoms of intravascular hemolysis as PNH red blood cells, which lack regulatory proteins needed to prevent attack by the terminal complement pathway, are destroyed intravascularly [1,14]. When the intravascular hemolysis of PNH has been controlled with C5 inhibitors (e.g., eculizumab and ravulizumab), extravascular hemolysis in the liver and spleen can develop as terminal complement pathway blockade increases the availability of C3b-opsonized PNH red blood cells and targets them for extravascular hemolysis [1,3,14]. This extravascular hemolysis can contribute to suboptimal hemoglobin levels and a continued need for transfusions in patients with PNH who receive C5 inhibitors [3,5,15,16,17].

The C3 inhibitor pegcetacoplan is the first PNH treatment aimed at an upstream complement pathway target [7,10,18]. It can provide comprehensive hemolysis control in C5 inhibitor-naïve patients with PNH and in those who remain anemic despite treatment with C5 inhibitors [10,11,19].

The 2021 FDA approval of pegcetacoplan for adults with PNH was primarily based on data from the PEGASUS trial (NCT03500549) [20], a phase 3, randomized, active-controlled, open-label study that compared the safety and efficacy of pegcetacoplan with that of eculizumab in adults with PNH who had a suboptimal response to eculizumab treatment [10]. Response was assessed by hematologic and clinical end points, including hemoglobin and lactate dehydrogenase levels, absolute reticulocyte counts, and transfusion independence, some of which were selected based on historical clinical trials in PNH [10,16].

Although these end points were sufficient for short-term clinical trials, a composite of multiple outcomes could provide a comprehensive response assessment for use in clinical practice [3]. To address this need, The European Society for Blood and Marrow Transplantation (EBMT) developed a consensus composite measure to classify the responses of complement inhibitor-treated patients with PNH [3]. In the absence of clinical guidelines for PNH treatment, the EBMT composite measure provides an objective and thorough assessment of treatment response.

Risitano, who authored the original EBMT classification criteria [3], applied the EBMT composite measure to the results of the PEGASUS trial [3]. Patient-level data of those who received either pegcetacoplan or eculizumab during the 16-week randomized controlled period were individually classified using the EBMT categories to compare hematologic responses [21].

Leveraging the analysis by Risitano and colleagues provides a unique opportunity to analyze the costs of switching from eculizumab to pegcetacoplan for patients with a limited response to eculizumab. The objective of the current analysis was to determine the average and incremental costs per responder for patients with PNH who had a prior incomplete response to eculizumab and switched to pegcetacoplan compared to those of patients who continued receiving eculizumab despite their incomplete responses. We undertook several scenario analyses, including costs-per-responder analyses of C5 inhibitor-naïve patients who received pegcetacoplan in other clinical trials and C5 inhibitor-naïve patients who received eculizumab in a real-world setting, to compare the findings to other populations.

## 2. Materials and Methods

Costs per responder were assessed for pegcetacoplan and eculizumab using data from post hoc EBMT response categorization analyses of the PEGASUS [21,22], PADDOCK and PALOMINO [22], and PRINCE [23] clinical trials and a real-world cohort study [24], plus drug and administration costs from REDBOOK [25,26,27]. This analysis was performed in Microsoft Excel 365 (Microsoft Corporation) and followed the best practice recommendations of the Second Panel on Cost-Effectiveness in Health and Medicine [28]. Institutional review board approval was not required because all data used were de-identified. All clinical trial patients provided written informed consent [10,11,19,29]. Consent was not needed for the de-identified data from other published studies and REDBOOK [24,25,26,27].

### 2.1. Trial Study Design and Patients

PEGASUS trial details have been published previously [10,20]. The trial included adults with PNH (confirmed by high-sensitivity flow cytometry) who had a suboptimal response to prior eculizumab treatment (i.e., hemoglobin < 105 g/L despite receiving stable dosages of eculizumab for ≥3 months before screening).

**Figure 1:**
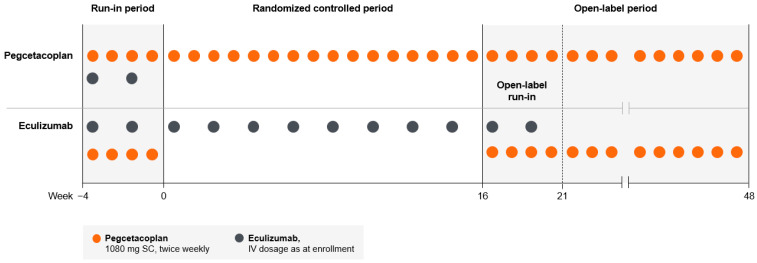
PEGASUS study design. IV, intravenous; SC, subcutaneous.

During the 4-week run-in period, all patients received self-administered SC pegcetacoplan 1080 mg twice weekly while continuing their current IV eculizumab dosages to maintain sufficient complement inhibition while pegcetacoplan was introduced [10,19]. After the run-in phase, patients were randomized (1:1) to monotherapy with eculizumab (N = 39) or pegcetacoplan (N = 41) for the 16-week randomized controlled period (Weeks 0–16). The randomized controlled period was followed by a 32-week open-label period (Weeks 16–48), during which patients who received eculizumab monotherapy in the randomized controlled period received eculizumab and pegcetacoplan during the 4-week open-label run-in period and then received pegcetacoplan monotherapy through Week 48, and patients who received pegcetacoplan monotherapy during the randomized controlled period continued to receive pegcetacoplan through Week 48 (Figure 1) [10].

### 2.2. Hematologic Response Categories

Methods for analyzing treatment responses during the randomized period of PEGASUS have been published [21]. In summary, PNH experts reviewed the PEGASUS trial data to classify patients into the following EBMT response categories: *complete response* (i.e., patients require no transfusions, have stable hemoglobin levels in the normal range, and have no evidence of hemolysis), *major response* (i.e., patients require no transfusions, have normal hemoglobin levels, and have evidence of residual intravascular or extravascular hemolysis), *good response* (i.e., patients require no transfusions and have evidence of chronic mild anemia or hemolysis), *partial response* (i.e., patients have chronic moderate anemia and/or require occasional transfusions of <3 units/6 months), *minor response* (i.e., patients require regular transfusions of 3–6 units/6 months), and *no response* (i.e., patients require regular and frequent transfusions of >6 units/6 months) [3,21]. Response category inputs from the previous analysis of the 16-week randomized controlled period of PEGASUS are shown in Table 1 [21].

### 2.3. Drug Acquisition and Administration Costs

Costs during the randomized period of PEGASUS were calculated from the beginning of PEGASUS through Week 16, including the run-in period. Trial-based dosages rather than label-based dosages were used as the base case to account for the common usage of increased drug dosages for patients not fully responding to treatment or experiencing breakthrough hemolysis. In a 2021 claims data analysis of US patients receiving eculizumab for PNH, only 29% of patients were receiving the label-recommended dosage [12]. The base case analysis used US drug acquisition and administration costs based on dosing regimens from FDA-approved prescribing information and the dosage observed in the PEGASUS trial, which included increased doses and shorter dosing intervals [18,30]. Scenario analyses using lower drug costs based on label-based dosing (i.e., not accounting for increased dosages) were also conducted to provide a conservative estimate of costs. All costs were undiscounted and are reported in 2020 US dollars.

Drug acquisition costs were based on the following 2021 wholesale acquisition costs [25]:

- Pegcetacoplan: USD 4403.84 for 1080 mg/20 mL
- Eculizumab: USD 6523.00 per 300 mg/30 mL

For pegcetacoplan, run-in costs included the 4-week run-in dosage of SC pegcetacoplan 1080 mg twice per week plus a continuation of the patient’s previous eculizumab dosage. After 4 weeks, the recommended pegcetacoplan maintenance dosage was 1080 mg twice per week. During the 16-week randomized controlled period of PEGASUS, nearly 5.0% of patients received an increased dosage of 1080 mg every 3 days (high maintenance dosage; Table 2) [10,18].

For eculizumab, the recommended maintenance dosage was 900 mg every 2 weeks [30]. Based on baseline PEGASUS eculizumab dosage data, 27.5% of patients received a first dosage escalation to 1200 mg every 2 weeks, and 2.5% received a second dosage escalation to 1500 mg every 2 weeks [10]. Four-week costs by treatment were calculated accordingly (Table 2) [10,18].

The administration unit costs of applicable treatment components (e.g., home infusion, clinic infusion, self-infusion pump) are shown (Table 3). Pegcetacoplan is self-administered as an SC infusion with a commercially available infusion pump. In the base case, the cost of the infusion pump to a health plan was assumed to be USD 0 (Table 3) [26,31], which is the most common scenario in the United States [27]. In the 4-week run-in of the PEGASUS trial, pegcetacoplan was administered with eculizumab to minimize the risk of hemolysis with abrupt eculizumab discontinuation [10]. The expected maintenance dose costs for home/clinic infusions of eculizumab were included in the costs to administer pegcetacoplan. A one-time cost for training by a health care professional (e.g., a nurse) in SC infusion was included, and pegcetacoplan was self-administered thereafter [18].

The calculated trial-based cost inputs for the base case analysis and the label-based cost inputs for the scenario analysis are summarized (Table 4) [26]. To reflect real-world treatment, costs during the run-in (i.e., the 4-week period in which patients received pegcetacoplan while continuing their eculizumab dosage) were only included in the pegcetacoplan treatment arm (not in the eculizumab arm). The calculated run-in cost for administering pegcetacoplan (including the coadministration of eculizumab) during the first month was approximately USD 965; the administration cost for maintenance therapy during the first month was USD 0 (Table 4). The administration costs for IV infusions of eculizumab were sourced from the published literature [26] and were calculated from the unit costs presented in Table 3 [26,31]. The calculated administration cost for maintenance eculizumab during that 4-week period was approximately USD 950 (Table 4).

### 2.4. Cost Analyses

The following primary outcomes were calculated for pegcetacoplan and eculizumab: average drug costs per patient over 16 weeks, average drug costs per patient per week, average drug costs per responder (i.e., complete, good, good to complete, and responder of any type), and percentage of total drug costs spent on patients with partial-to-no response or those with discontinued/missing status.

The average drug costs by response category were calculated as mean drug costs per treated patient over 16 weeks divided by the percentage of patients with the stated EBMT response category. The incremental costs per incremental responder at 16 weeks for a given response level were calculated as follows:

$$\frac{Pegcetacoplan\;mean\;costs\;per\;patient\;over\;16\;weeks-eculizumab\;mean\;costs\;per\;patient\;over\;16\;weeks}{Pegcetacoplan\;\%\;with\;given\;response\;at\;16\;weeks-eculizumab\;\%\;with\;given\;response\;at\;16\;weeks}$$

The number needed to treat (NNT) to achieve each level of response (compared with no treatment) was calculated at 16 weeks as the reciprocal of the percentage of patients with a given response for a given treatment.

### 2.5. Scenario Analyses

An initial scenario analysis was conducted, excluding administration costs, to compare the average costs per response from a pharmacy cost perspective. A second scenario was conducted using the label dosages for pegcetacoplan and eculizumab, without accounting for increased dosage, to provide a conservative estimate that does not account for real-world treatment variations.

Additional scenario analyses were performed using all available data from recent PNH clinical trial response categorization studies (as of July 2022) [21,22,23,24] to estimate the average costs per treated patient, average costs per good-to-complete responder (i.e., patient with a good, major, or complete response), percentage of total costs for patients with partial-to-no response (i.e., patients with a partial, minor, or no response) or discontinued/missing status, and NNT for pegcetacoplan and eculizumab across a range of PNH patient populations. These additional analyses include the following:

1. Calculation of the average drug costs per responder at Week 48 for patients with a suboptimal response to prior eculizumab treatment with the use of categorized response data for those treated with pegcetacoplan from both the randomized controlled period (Weeks 0–16) and the open-label period (Weeks 16–48) of the PEGASUS trial [21,22] (Supplementary Materials Section S1).
2. Calculation of the average drug costs per responder at Weeks 16 and 48 for C5 inhibitor-naïve patients (i.e., PNH patients who had not previously received treatment with the C5 inhibitors eculizumab or ravulizumab) who initiated pegcetacoplan treatment with the use of nonrandomized, categorized response data from the phase 1b PADDOCK and the phase 2a PALOMINO trials [22] (Supplementary Materials Section S2).
3. Calculation of the average drug costs per responder at Week 26 for C5 inhibitor-naïve (i.e., had not received C5 inhibitors within 3 months of screening) patients initiating pegcetacoplan treatment with the use of nonrandomized, categorized response data from the phase 3 PRINCE trial [23] (Supplementary Materials Section S2).
4. Calculation of the average drug costs per responder at Months 6 and 12 with the use of categorized, real-world response data from C5 inhibitor-naïve patients initiating eculizumab treatment [24] (Supplementary Materials Section S3).

## 3. Results

### 3.1. Base Case Analysis

Baseline characteristics were generally balanced between the pegcetacoplan (N = 41) and eculizumab (N = 39) arms of PEGASUS [10]. Over the 16-week, randomized controlled period, the average drug cost per patient was USD 186,411 with pegcetacoplan and USD 177,313 with eculizumab (the latter included the run-in drug costs for eculizumab) (Table 5). The average 16-week drug cost per patient with a complete response was USD 477,679 for those treated with pegcetacoplan; this value was not calculable for the eculizumab group because no patients had a complete response. The NNT per complete responder was 2.6 with pegcetacoplan and was not calculable with eculizumab. The average drug costs per responder and the NNTs were much lower with pegcetacoplan than with eculizumab in the good and the good-to-complete response categories. The incremental cost for pegcetacoplan (vs. eculizumab) per additional complete responder was USD 23,315. The incremental drug costs for the other response categories are shown (Table 5).

Total costs for patients with partial-to-no responses during 16 weeks of treatment were USD 2.0 million for pegcetacoplan and USD 6.5 million for eculizumab (Table 5). In the pegcetacoplan group, 27% of the total drug costs were spent on patients with partial-to-no response or those with discontinued/missing status; in the eculizumab group, these patients accounted for 95% of the total drug costs.

### 3.2. Scenario Analyses Results

Across all drug cost scenarios with and without administration costs and by dosage, the average costs per responder remained lower for pegcetacoplan than for eculizumab for good-to-complete responders and for partial-to-no responders over label and trial dosages and when excluding administration costs (Supplementary Materials Section S4).

The results of additional scenario analyses for patients with good-to-complete response and partial-to-no response in alternative PNH populations and with additional treatment durations using all available data from recent categorization studies of PNH response are shown in Table 6 [21,22,23,24]. For patients with a suboptimal response to eculizumab who switched to pegcetacoplan in PEGASUS, the average drug cost per good-to-complete responder was somewhat lower at Week 48 (USD 15,459 per week) than at Week 16 (USD 15,923 per week, the base case analysis). The NNT per good-to-complete responder was 1.6 at Week 48 and 1.4 at Week 16 (Table 6) [21].

For C5 inhibitor-naïve patients treated with pegcetacoplan, the average drug costs per good-to-complete responder were lower in both the PADDOCK/PALOMINO trials and the PRINCE trial compared with the base case population from PEGASUS at all time points (Table 6) [21,22,23]. In the PADDOCK/PALOMINO group, the average weekly drug costs per good-to-complete responder were USD 11,857 at Week 16 and USD 14,227 at Week 48 [22]. In the PRINCE group, the average weekly drug cost per good-to-complete responder was USD 11,115 at Week 26 [23]. The NNTs per good-to-complete responder were 1.3 and 1.6 at Weeks 16 and 48, respectively, for PADDOCK/PALOMINO and 1.3 for Week 26 of PRINCE.

For a real-world cohort of C5 inhibitor-naïve patients treated with eculizumab, the average drug costs per good-to-complete responder were lower (USD 23,455 per week at 6 months; USD 19,273 per week at 1 year) than that for the base case, eculizumab-treated population from PEGASUS (USD 216,100 per week at 16 weeks; Table 6) [21,24]. The average drug costs per good-to-complete responder, percentage of total costs spent on patients with partial-to-no response, and NNT were higher for eculizumab based on data from a real-world cohort than for any pegcetacoplan-treated population over any treatment duration.

## 4. Discussion

The 16-week randomized controlled period of PEGASUS was the first head-to-head comparison of pegcetacoplan vs. eculizumab for the treatment of patients with PNH who had a suboptimal response to eculizumab [10]. The current analysis shows that, among these patients, switching to pegcetacoplan treatment can result in a better response than continuing eculizumab treatment at a weekly cost of USD 11,651 (pegcetacoplan) vs. USD 11,082 (eculizumab). Despite higher average drug costs with pegcetacoplan, the NNTs and average costs over 16 weeks per complete, good, and good-to-complete responders were lower for patients who switched to pegcetacoplan than for those who continued eculizumab.

Pegcetacoplan treatment allowed participants with a prior suboptimal response to eculizumab to have a complete response according to the EBMT criteria, while none of the patients who continued eculizumab treatment had a complete response [21]. A greater percentage of patients who switched to pegcetacoplan had a good-to-complete EBMT response (i.e., 73% with pegcetacoplan vs. 5% with eculizumab). The incremental cost per additional good-to-complete responder over 16 weeks was USD 13,372. This means that, on average, spending an additional USD 13,372 over 16 weeks, which is equivalent to increasing total per-patient costs by 8.0%, on pegcetacoplan rather than on eculizumab would result in one additional patient with a good-to-complete response. The NNT per good-to-complete responder was 1.4 for pegcetacoplan and 19.5 for eculizumab; generally, a higher NNT indicates that a treatment is less effective [32]. All results are specific to the PEGASUS patient population, which was selected based on a prior suboptimal response to eculizumab. Thus, the results of our base case analysis of the PEGASUS trial should not be generalized beyond this patient population and do not apply to eculizumab-naïve patients.

Given that the study population was limited to patients with a suboptimal eculizumab response, scenario analyses were conducted for additional PNH populations and treatment durations: at Week 48 for patients with a suboptimal response to prior eculizumab therapy treated with pegcetacoplan in PEGASUS (the base case population); at Weeks 16, 26, and 48 for C5 inhibitor-naïve patients initiating pegcetacoplan treatment in clinical trials; and at 6 months and 1 year for a real-world cohort of C5 inhibitor-naïve patients initiating eculizumab treatment. Average drug costs per good-to-complete response per week for pegcetacoplan were similar across all patient populations and treatment durations, except the base case, in which the average costs were higher. Higher costs in the base case population were expected because these patients had prior suboptimal responses to eculizumab.

The scenario results for eculizumab based on real-world data show a higher rate of response to eculizumab than the rate of response observed in the PEGASUS trial data. These results were expected, as the real-world data are from C5 inhibitor-naïve patients initiating eculizumab treatment rather than from patients with a suboptimal response to eculizumab. Despite C5 inhibitor-naïve patients from a real-world cohort having a markedly higher eculizumab response rate and substantially lower drug costs per responder than patients from PEGASUS, the average drug costs per responder, percentage of total costs spent on patients with partial-to-no response, and NNT remained higher for eculizumab-treated patients than for any pegcetacoplan-treated population. The eculizumab response in the real-world data may be a best-case scenario for eculizumab, as the 30% to 61% of patients who discontinued eculizumab in the first 18 months of treatment were excluded [12,33]. These scenario results should be interpreted with caution because they come from multiple uncontrolled studies with different inclusion and exclusion criteria. Long-term head-to-head studies of the costs and effectiveness of pegcetacoplan and eculizumab in representative PNH populations are needed.

### 4.1. Study Limitations

This analysis has several limitations. First, it did not include the C5 inhibitor ravulizumab due to a lack of head-to-head data for all available complement inhibitor treatments. To our knowledge, there are no published analyses categorizing patients treated with ravulizumab based on EBMT hematologic response. Second, the populations studied were small because PNH is a rare disease, with an estimated 5000–6000 prevalent cases in the US [34]. Third, this analysis was conducted from the cost perspectives of a third-party payer and does not capture other treatment-related costs (e.g., transfusions, adverse events) and important differences in treatment factors that may affect patients’ quality of life (e.g., drug administration convenience, treatment frequency). Lost productivity due to PNH, which is estimated to average USD 4.3 million when modeled over a 2-year period in the United States, is also not captured [35]. Cost-effectiveness analyses from a societal perspective are important to compare the full economic benefits of PNH treatment. Fourth, this analysis does not include run-in dosing costs for patients switching from ravulizumab to pegcetacoplan, which would likely differ from the run-in dosing costs presented here. Fifth, patient-level-linked data for drug dosage and treatment response were not available for all studies, nor would the studies have been statistically powered for such an analysis. Consequently, average drug costs by drug, dosage, and trial were used rather than drug costs by patient or treatment response level. Sixth, patients in the PEGASUS trial had a prior suboptimal response to eculizumab and were naïve to pegcetacoplan; as a result, head-to-head costs per responder results should not be generalized to patients naïve to eculizumab. To address this, results for a real-world, eculizumab-treated population without a prior suboptimal response to eculizumab are presented. These results confirm, as expected, lower average costs per responder for C5 inhibitor-naïve patients. This confirmation does not detract from the study’s findings, but reiterates that the findings should not be generalized to eculizumab-naïve patients. Finally, costs used in our analysis are publicly available wholesale acquisition costs. These may differ from prices encountered by health care providers through contracting and discounting programs, the details of which are not publicly available. The results of this analysis are specific to the PEGASUS population and are based on data from a clinical trial setting, which limits their generalizability.

### 4.2. Study Strengths

The strengths of this analysis include the use of all available EBMT response categorization studies (as of July 2022) in the scenario analyses. These analyses showed similar average drug costs per response for pegcetacoplan across study populations with different treatment histories and baseline disease severities. Although the scenario analyses are based on uncontrolled (i.e., one arm) trial data for pegcetacoplan, the results suggest that our estimated average drug costs and average drug costs per response for pegcetacoplan are robust, at least for the first year of treatment. By using the EBMT consensus classifications to assess clinical response with a composite set of PNH-relevant measures, these results may inform payers of cost-efficient choices. Payers, physicians, and patients have become more interested in treatment costs and cost-effectiveness, particularly for chronic conditions such as PNH.

## 5. Conclusions

This analysis of PEGASUS data showed that switching from eculizumab to pegcetacoplan is a cost-effective approach to improve responses in patients with PNH who have had a suboptimal response to eculizumab. Treatment of PNH with pegcetacoplan, a C3-targeted therapy, allowed more patients to have an EBMT-defined, good-to-complete response, and drug costs per patient achieving a good-to-complete response were substantially lower for those treated with pegcetacoplan. These results are applicable to patients with a suboptimal response to prior eculizumab treatment only. Scenario analysis results suggest avenues for future research.

## Figures and Tables

**Table 1:** EBMT hematologic responses to pegcetacoplan and eculizumab in patients with a suboptimal response to prior eculizumab treatment.

	PEGASUS Trial ^a,b^	
	Pegcetacoplan N = 41 16 Weeks	Eculizumab N = 39 16 Weeks
Individual response categories, n (%)		
Complete responders	16 (39)	0
Major responders	0	0
Good responders	14 (34)	2 (5)
Partial responders	6 (15)	15 (38)
Minor responders	2 (5)	13 (33)
Nonresponders	0	9 (23)
Discontinued or missing	3 (7)	0
Combined response categories, n (%)		
Good-to-complete responders	30 (73)	2 (5)
Partial-to-no responders or discontinued/missing	11 (27)	37 (95)
Any response	38 (93)	30 (77)

EBMT, The European Society for Blood and Marrow Transplantation. ^a^ Eligibility for the PEGASUS trial required patients to have a suboptimal response to ≥3 months of stable dosing and consistent eculizumab treatment, with *suboptimal response* defined as hemoglobin levels < 105 g/L. For the results for other patient populations, see Table 6 and the Supplementary Materials. ^b^ Source: Risitano et al. [21].

**Table 2:** Calculated weighted average 4-week drug acquisition costs of pegcetacoplan and eculizumab at varying dosages.

Treatment	Dosage	Percentage Receiving Dosage	4-Week Drug Acquisition Costs ^b^	Source
*Pegcetacoplan*				
Maintenance dose	1080 mg twice per week	95.1%	USD 35,231	Apellis Pharmaceuticals, Inc. [18]
High maintenance dose	1080 mg every third day	4.9%	USD 41,103	Apellis Pharmaceuticals, Inc. [18]
Maintenance dose ^a^	Weighted average used in model ^a^	Not applicable	USD 35,517 ^a^	Calculated ^a^
*Eculizumab*				
Maintenance dose	900 mg every 2 weeks	70.0%	USD 39,138	Hillmen et al. [10]
First dose increase	1200 mg every 2 weeks	27.5%	USD 52,184	Hillmen et al. [10]
Second dose increase	1500 mg every 2 weeks	2.5%	USD 65,230	Hillmen et al. [10]
Maintenance dose ^a^	Weighted average used in model ^a^	Not applicable	USD 43,378 ^a^	Calculated ^a^

^a^ Values are the inputs used in the model. ^b^ All costs are reported in 2020 US dollars.

**Table 3:** Administration unit costs of various treatment components.

Treatment	Unit Costs ^b^	Source
Home infusion (50% of infusions for eculizumab)	USD 261	Tomazos et al. [26]
Clinic infusion (50% of infusions for eculizumab)	USD 689	Tomazos et al. [26]
Self-infusion pump ^a^	USD 0	Assumed
Therapeutic, prophylactic, or diagnostic injection SC or intramuscular	USD 14	Centers for Medicare & Medicaid Services [31] (CPT 96372)

CPT, Current Procedural Terminology; N/A, not applicable; SC, subcutaneous. ^a^ A patient’s health care plan determines whether there are costs associated with the self-infusion pump. ^b^ All costs are reported in 2020 US dollars.

**Table 4:** Total 4-week drug acquisition and administration costs by treatment.

Dosage	Pegcetacoplan ^b^	Eculizumab ^b^
Trial dosage (base case analysis) ^a^		
Drug acquisition		
Run-in	USD 78,895.10 ^c^	Not applicable
Maintenance	USD 35,517.15	USD 43,377.95
Drug administration		
Run-in	USD 964.65 ^c^	Not applicable
Maintenance	USD 0.00	USD 950.21
Label dosage (scenario analysis)		
Drug acquisition		
Run-in	USD 74,368.72 ^c^	Not applicable
Maintenance	USD 35,230.72	USD 39,138.00
Drug administration		
Run-in	USD 964.65 ^c^	Not applicable
Maintenance	USD 0.00	USD 950.21

^a^ Accounts for increased dosage and shorter dosing intervals observed in the clinical trial. ^b^ All costs are reported in 2020 US dollars. ^c^ Costs during the run-in (i.e., the period in which patients received pegcetacoplan while continuing their prior eculizumab dosage for 4 weeks) were only included in the pegcetacoplan treatment arm (not in the eculizumab arm). The run-in drug and administration costs for pegcetacoplan included the 4-week run-in dosage of 1080 mg twice per week of pegcetacoplan plus continuation of eculizumab dosage as at enrollment (USD 35,517 + USD 43,378).

**Table 5:** Costs ^a^ per responder results for patients with PNH with a suboptimal response to prior eculizumab treatment.

	PEGASUS Trial ^e,f^	
	Pegcetacoplan 16 Weeks N = 41	Eculizumab 16 Weeks N = 39
Average drug costs per patient over 16 weeks	USD 186,411	USD 177,313
Average drug costs per patient per week ^b^	USD 11,651	USD 11,082
Total drug costs for patients with partial-to-no response or discontinued/missing status (percentage of total costs)	USD 2,010,386 (27)	USD 6,530,630 (95)
Drug costs by EBMT response category over 16 weeks		
Average drug costs per complete responder ^c^	USD 477,679	Not applicable, no complete responders
	(USD 29,855 per week)	
Average drug costs per good responder ^c^	USD 545,919	USD 3,457,597
	(USD 34,120 per week)	(USD 216,100 per week)
Average drug costs per good-to-complete responder ^c^	USD 254,762	USD 3,457,597
	(USD 15,923 per week)	(USD 216,100 per week)
Average drug costs per responder of any type ^c^	USD 201,128	USD 230,506
	(USD 12,570 per week)	(USD 14,407 per week)
NNT by EBMT response category (percentage with response)		
NNT per complete responder	2.6 (39)	Not applicable, no complete responders (0)
NNT per good responder	2.9 (34)	19.5 (5)
NNT per good-to-complete responder	1.4 (73)	19.5 (5)
NNT per responder (of any type)	1.1 (93)	1.3 (77)
Incremental drug costs per responder by category	Pegcetacoplan minus eculizumab ^d^	
Incremental drug costs per additional complete responder ^d^	USD 23,315	
Incremental drug costs per additional good responder ^d^	USD 31,355	
Incremental drug costs per additional good-to-complete responder ^d^	USD 13,372	
Incremental drug costs per additional responder (of any type) ^d^	USD 57,732	

EBMT, The European Society for Blood and Marrow Transplantation; NNT, number needed to treat; PNH, paroxysmal nocturnal hemoglobinuria. ^a^ All costs are reported in 2020 US dollars. ^b^ Average drug costs per treated patient per week = weekly administered dose in the PEGASUS trial for each patient × (drug + administration costs); this includes dosage and frequency escalations. ^c^ Costs per response category = mean drug costs per treated patient over time period/percentage of patients with the stated EBMT response category. Also calculated are the average costs per week per responder (in parenthesis). ^d^ Incremental drug costs per responder = (mean costs for pegcetacoplan over 16 weeks—mean costs for eculizumab over 16 weeks)/(percentage with given response for pegcetacoplan at 16 weeks − percentage with given response for eculizumab at 16 weeks). A positive result in this case indicates increased costs associated with pegcetacoplan and improved response. ^e^ Eligibility for the PEGASUS trial required patients to have a suboptimal response to ≥3 months of stable dosing and consistent eculizumab treatment, with *suboptimal response* defined as hemoglobin levels < 105 g/L. For the results for other patient populations, see Table 6 and the Supplementary Materials. ^f^ Source: Risitano et al. [21].

**Table 6:** EBMT response criteria and key results for pegcetacoplan and eculizumab across all populations and treatment durations studied.

	Pegcetacoplan					Eculizumab		
	PEGASUS ^d^ N = 41		PADDOCK/ PALOMINO ^e^ N = 24		PRINCE ^f^ N = 35	PEGASUS ^d^ N = 39	Real-World Data ^g^ Debureaux et al. N = 127	
	Suboptimal Response to Prior Eculizumab Treatment		C5 Inhibitor Naive		C5 Inhibitor Naive	Suboptimal Response to Prior Eculizumab Treatment	C5 Inhibitor Naive	
	16 Weeks	48 Weeks	16 Weeks	48 Weeks	26 Weeks	16 Weeks	6 Months	12 Months
*EBMT combined response category*								
Good-to-complete responders, n (%)	30 (73)	26 (63)	18 (75)	15 (63)	28 (80)	2 (5)	N/A (47)	N/A (58)
Partial-to-no response or discontinued/missing, n (%)	11 (27)	15 (37)	6 (25)	9 (38)	7 (20)	37 (95)	N/A (53)	N/A (43)
*Key costs per responder results ^a^*								
Average drug costs per patient per week ^b^	USD 11,651	USD 9803	USD 8892	USD 8892	USD 8892	USD 11,082	USD 11,082	USD 11,082
Average drug costs per good-to-complete responder over study time period ^c^	USD 254,762	USD 742,019	USD 189,706	USD 682,895	USD 288,994	USD 3,457,597	USD 611,483	USD 1,004,958
Average drug costs per good-to-complete responder per week ^c^	USD 15,923	USD 15,459	USD 11,857	USD 14,227	USD 11,115	USD 216,100	USD 23,455	USD 19,273
Percentage of total drug costs for partial-to-no response or discontinued/missing	27	37	25	38	20	95	53	43
NNT per good-to-complete responder	1.4	1.6	1.3	1.6	1.3	19.5	2.1	1.7

*C5,* complement component 5; *EBMT*, The European Society for Blood and Marrow Transplantation; *N/A*, not available; *NNT*, number needed to treat. ^a^ All costs are reported in 2020 US dollars. ^b^ Average drug costs per treated patient per week = weekly administered dose for each patient × (drug + administration costs); this includes dosage and frequency escalations. ^c^ Costs per response category = mean drug costs per treated patient over time period/percentage of patients with the stated EBMT response. ^d^ Sources: Risitano et al. [21,22]. ^e^ Source: Risitano et al. [22]. ^f^ Source: Risitano et al. [23]. ^g^ Source: Debureaux et al. [24].

## Data Availability

Pegcetacoplan trial data used for this study are from Apellis Pharmaceuticals, Inc., and were used under license for the current study. Therefore, restrictions apply to the availability of these data, which cannot be made publicly available. Access to this dataset may be granted to other interested parties by Apellis Pharmaceuticals, Inc., but may be subject to limitations based on how the original clinical trial data were acquired. Other data used in this study are publicly available.

## Supplementary Materials

The following supporting information can be downloaded at https://www.mdpi.com/article/10.3390/hematolrep15040060/s1, Supplementary Materials Section S1: Analysis of 48-week data for patients with a suboptimal response to prior eculizumab treatment; 1.1 Description of the 48-week analysis of PEGASUS; Table S1: EBMT hematologic response at 48 weeks for patients on pegcetacoplan with a suboptimal response to prior eculizumab treatment; Supplementary Materials Section S2: Pegcetacoplan treatment in C5 inhibitor-naïve patients; 2.1 Study design and patients; 2.2 Study dosing and drug acquisition and administration costs; Table S2: Calculated weighted average of 4-week drug acquisition costs; 2.3 Hematologic response; Table S3: EBMT hematologic response for C5 inhibitor-naïve patients; Supplementary Materials Section S3. Real-world data for C5 inhibitor-naïve patients initiating eculizumab treatment; 3.1 Study design and patients; 3.2 Study dosing and drug acquisition and administration costs; 3.3 Hematologic response; Table S4: EBMT hematologic response for a real-world cohort of C5 inhibitor-naïve patients initiating eculizumab treatment; Supplementary Materials Section S4. Scenario analyses of drug costs with and without administration costs and by dosage; 4.1 Scenario descriptions; Table S5: Drug costs with and without administration costs and by dosage.
