# Supplementary material for: Analysis of Costs per Responder in US Adults with Paroxysmal Nocturnal Hemoglobinuria with a Suboptimal Response to Prior Eculizumab Treatment
Source: Hematol Rep. 2023 Oct 13;15(4):578–91. doi: 10.3390/hematolrep15040060 (PMC10594490; doi:10.3390/hematolrep15040060)
Supplement: Supplementary file 1 [file hematolrep-15-00060-s001.zip › hematolrep-2494404-supplementary.pdf]

**Supplementary Materials**

**Analysis of Costs per Responder in US Adults with Paroxysmal Nocturnal Hemoglobinuria with a Suboptimal Response to Prior Eculizumab Treatment**


| Table of Contents | Page(s) |
|---|---|
| **Section S1. Analysis of 48-week data for patients with a suboptimal response to prior eculizumab treatment** | **2** |
| 1.1 Description of the 48-week analysis of PEGASUS | 2 |
| Table S1. EBMT hematologic response at 48 weeks for patients on pegcetacoplan with a suboptimal response to prior eculizumab treatment | 2 |
| **Section S2. Pegcetacoplan treatment in C5 inhibitor–naive patients** | **3-4** |
| 2.1 Study design and patients | 3 |
| 2.2 Study dosing and drug acquisition and administration costs | 3 |
| Table S2. Calculated weighted average of 4-week drug acquisition costs | 3-4 |
| 2.3 Hematologic response | 4 |
| Table S3. EBMT hematologic response for C5 inhibitor–naive patients | 4 |
| **Section S3. Real-world data for C5 inhibitor–naive patients initiating eculizumab treatment** | **5-6** |
| 3.1 Study design and patients | 5 |
| 3.2 Study dosing and drug acquisition and administration costs | 5 |
| 3.3 Hematologic response | 5 |
| Table S4. EBMT hematologic response for a real-world cohort of C5 inhibitor–naive patients initiating eculizumab treatment | 6 |
| **Section S4. Scenario analyses of drug costs with and without administration costs and by dosage** | **7** |
| 4.1 Scenario descriptions | 7 |
| Table S5. Drug costs with and without administration costs and by dosage | 7 |
| **References** | **8** |

## Section S1. Analysis of 48-week data for patients with a suboptimal response to prior eculizumab treatment

### 1.1 Description of the 48-week analysis of PEGASUS

This analysis of the PEGASUS trial followed patients with paroxysmal nocturnal hemoglobinuria (PNH) who received pegcetacoplan for an additional 32 weeks after the 16-week, randomized controlled trial period. These additional 32 weeks of follow-up did not include an eculizumab comparison arm; inputs and results are presented here for pegcetacoplan alone at 48 weeks of follow-up (**Table S1**) [1].

**Table S1.** EBMT hematologic responses at 48 weeks for patients on pegcetacoplan with a suboptimal response to prior eculizumab treatment.

| **Pegcetacoplan** | **PEGASUS Trial**[a] **N=41 48 Weeks** |
|---|---|
| *Individual response categories, n (%)* | |
| Complete responders | 11 (27) |
| Major responders | 2 (5) |
| Good responders | 13 (32) |
| Partial responders | 6 (15) |
| Minor responders | 3 (7) |
| Nonresponders | 0 |
| Discontinued or missing | 6 (15) |
| *Combined response categories, n (%)* | |
| Good-to-complete responders | 26 (63) |
| Partial-to-no responders or discontinued/missing | 15 (37) |

*EBMT*, European Society for Blood and Marrow Transplantation.

[a]Source: Risitano et al. [1]

## Section S2. Pegcetacoplan treatment in C5 inhibitor–naive patients

### 2.1 Study design and patients

Analyses of patient-level data from the open-label PADDOCK (NCT02588833), PALOMINO (NCT03593200), and PRINCE (NCT04085601) trials were conducted by Risitano and colleagues [1,2] using the European Society for Blood and Marrow Transplantation (EBMT) response criteria to provide supplementary data on the cost per response for C5 inhibitor–naive patients initiating pegcetacoplan treatment.

The analysis of the PADDOCK (phase 1b, 2 cohorts, n=20) and PALOMINO (phase 2a, 1 cohort, n=4) trial data was conducted for patients aged ≥18 years who had not previously been treated with C5 inhibitors and were diagnosed with PNH [1,3]. More information on inclusion and exclusion criteria is available in the publication by Wong and colleagues [3].

The analysis of the PRINCE (phase 3, 1 cohort, n=35) trial data included patients aged ≥18 years who were diagnosed with PNH and had not been treated with C5 inhibitors within 3 months of screening [2,4]. More information on inclusion and exclusion criteria is available in Wong et al [4].

### 2.2 Study dosing and drug acquisition and administration costs

C5 inhibitor–naive patients initiating pegcetacoplan received pegcetacoplan monotherapy. A one-time cost for training by a health care professional (eg, a nurse) in subcutaneous infusion was included, with pegcetacoplan self-administered thereafter [5]. The calculated costs to administer pegcetacoplan for the first month was $14 for C5 inhibitor–naive patients and $0 thereafter. Based on clinical trial data, 6% of C5 inhibitor–naive patients in the PRINCE trial received an increased dosage of 1080 mg every 3 days. This was used to calculate drug costs for pegcetacoplan for all cost-per-responder analyses of C5 inhibitor–naive patients. See **Table S2.**

**Table S2.** Calculated weighted average of 4-week drug acquisition costs.[a]

| Pegcetacoplan | Dosage | Percentage Receiving Dosage | 4-Week Cost | Source |
|---|---|---|---|---|
| Maintenance dose | 1080 mg twice per week | 94.3% | $35,231 | Hillmen et al. [5] |
| High maintenance dose | 1080 mg every third day | 5.7% | $41,103 | Hillmen et al. [5] |
| Maintenance dose[b] | Weighted average used in model[b] | Not applicable | $35,566[b] | Calculated[b] |

[a]All costs reported in 2020 US dollars.

[b]Values are the inputs used in the model.

### 2.3 Hematologic response

The number of patients in each category of hematologic response is provided in **Table S3**.

**Table S3.** EBMT hematologic response for C5 inhibitor–naive patients.

| Pegcetacoplan | PADDOCK/PALOMINO Trials[a] N=24 16 Weeks | PADDOCK/PALOMINO Trials[a] N=24 48 Weeks | PRINCE Trial[b] N=35 26 Weeks |
|---|---|---|---|
| *Individual response categories, n (%)* | | | |
| Complete responders | 11 (46) | 10 (42) | 18 (51) |
| Major responders | 0 | 2 (8) | 0 |
| Good responders | 7 (29) | 3 (13) | 10 (29) |
| Partial responders | 1 (4) | 5 (21) | 5 (14) |
| Minor responders | 2 (8) | 1 (4) | 1 (3) |
| Nonresponders | 1 (4) | 0 | 0 |
| Discontinued or missing | 2 (8) | 3 (13) | 1 (3) |
| *Combined response categories, n (%)* | | | |
| Good-to-complete responders | 18 (75) | 15 (63) | 28 (80) |
| Partial-to-no responders or discontinued/missing | 6 (25) | 9 (38) | 7 (20) |

*EBMT*, European Society for Blood and Marrow Transplantation.

[a]Source: Risitano et al. [1]

[b]Source: Risitano et al. [2]

## Section S3. Real-world data for C5 inhibitor–naive patients initiating eculizumab treatment

### 3.1 Study design and patients

The analysis of real-world data used patient-level clinical data from 6 international PNH treatment centers (N=160), although only data from patients with 18 months of follow-up and a complete record were analyzed (n=127). All patients were women, all were initiating eculizumab to treat hemolytic PNH, and their median age was 39 years (range, 12-90 years). The median duration of treatment with eculizumab was 5.8 years (range, 0.5-14.5 years), and 10 patients had less than 18 months of follow-up. More information on the cohort inclusion and exclusion criteria are available in Debureaux et al. [6]

### 3.2 Study dosing and drug acquisition and administration costs

At the last data timepoint, 83% of patients received the label-recommended dosage of 900 mg of eculizumab every 2 weeks. A further 16% received 1200 mg of eculizumab every 2 weeks, and 1% received 900 mg of eculizumab every 10-11 days. For calculating drug costs, it was assumed that 83% received 900 mg every 2 weeks and 17% received 1200 mg every 2 weeks, resulting in a 4-week cost of $41,356 and a 4-week administration cost of $950, which is identical to the 4-week costs for eculizumab tested in the base case.

### 3.3 Hematologic response

The percentage of patients in each category of hematologic response for a real-world cohort of C5 inhibitor–naive patients is given in **Table S4**. Counts were not reported in the publication and are thus unavailable.

**Table S4.** EBMT hematologic response for a real-world cohort of C5 inhibitor–naive patients initiating eculizumab treatment.[a,b]

| Eculizumab | n=127<br>6 Months | n=127<br>12 Months |
|---|---|---|
| *Individual response categories, %* | | |
| Complete responders | 7.1 | 11.8 |
| Major responders | 3.1 | 5.5 |
| Good responders | 37.0 | 40.2 |
| Partial responders | 33.1 | 31.5 |
| Minor responders | 15.7 | 7.9 |
| Nonresponders | 3.9 | 3.1 |
| Discontinued or missing | 0 | 0 |
| *Combined response categories, %* | | |
| Good-to-complete responders | 47.2 | 57.5 |
| Partial-to-no responders or discontinued/missing | 52.8 | 42.5 |

[a]Source: Debureaux et al. [6]

[b]Counts were not included because they were not reported in the source publication.

**Section S4. Scenario analyses of drug costs with and without administration costs and by dosage**

### 4.1 Scenario descriptions

Two scenario analyses of drug costs were conducted. The first excluded administration costs and the second used the label dosage for pegcetacoplan and eculizumab without accounting for dosage increases. Results are shown in **Table S5**.

**Table S5.** Drug costs with and without administration costs and by dosage.[a]

| | **Pegcetacoplan N=41 16 weeks** | **Eculizumab N=39 16 weeks** |
|---|---|---|
| *Base case: including drug acquisition and administration costs and trial-based dosage* | | |
| Average drug cost per good-to-complete responder | $254,762 | $3,457,597 |
| Total drug cost for patients with partial-to-no response or discontinued/missing status (percentage of total costs) | $2,050,523 (27) | $6,560,568 (95) |
| Incremental cost per additional good-to-complete responder | Pegcetacoplan minus eculizumab[b] $13,372 | |
| *Scenario 1: drug costs only (excluding administration costs)* | | |
| Average drug cost per good-to-complete responder | $253,444 | $3,383,480 |
| Total cost for patients with partial-to-no response or discontinued/missing status (percentage of total costs) | $2,039,912 (27) | $6,419,937 (95) |
| Incremental drug cost per additional good-to-complete responder | Pegcetacoplan minus eculizumab[b] $17,540 | |
| *Scenario 2: label dosage (excluding dose and dose frequency changes for pegcetacoplan and eculizumab)* | | |
| Average drug cost per good-to-complete responder | $247,402 | $3,126,881 |
| Total cost for patients with partial-to-no response or discontinued/missing status (percentage of total costs) | $1,991,281 (27) | $5,933,056 (95) |
| Incremental drug cost per additional good-to-complete responder | Pegcetacoplan minus eculizumab[b] $30,382 | |

[a]All costs are reported in 2020 US dollars.

[b]Incremental drug costs per responder = (mean costs for pegcetacoplan over 16 weeks – mean costs for eculizumab over 16 weeks) / (percentage with given response for pegcetacoplan at 16 weeks − percentage with given response for eculizumab at 16 weeks). A positive result in this case indicates increased costs associated with pegcetacoplan and improved response.

## References


1. Risitano, A.; Wong, R.S.; Al-Adhami, M.; Chen, C.; de Latour, R.P. Categorized hematologic response to pegcetacoplan and correlations with quality of life in patients with paroxysmal nocturnal hemoglobinuria: post hoc analysis of data from phase 1b, phase 2a, and phase 3 trials. *Blood* **2021**, *138* (Suppl. 1), 1104. doi:10.1182/blood-2021-147988
2. Risitano, A.; Wong, R.; Al-Adhami M.; Savage, J.; Horneff, R.; de Latour, R.P. P833: Categorizing hematological response to pegcetacoplan in patients with paroxysmal nocturnal hemoglobinuria: a post hoc analysis of the phase 3 PRINCE study data. *HemaSphere* **2022**, *6* (Suppl.), 727–728. doi:10.1097/01.HS9.0000846216.47601.df
3. Wong, R.S.M., Pullon, H.W.H., Amine, I., Bogdanovic, A.; Deschatelets, P.; Francois, C.G; Ignatova, K.; Issaragrisil, S.; Niparuck, P.; Numbenjapon, T.; et al. Inhibition of C3 with pegcetacoplan results in normalization of hemolysis markers in paroxysmal nocturnal hemoglobinuria. *Ann Hematol* **2022**, *101*, 1971–1986.
4. Wong, R.S.M.; Navarro-Cabrera, J.R.; Comia, N.S.; Goh, Y.T.; Idrobo, H.; Kongkabpan, D.; Gomez-Almaguer, D.; Al-Adhami, M.; Ajayi, T.; Alvarenga, P.; et al. Pegcetacoplan controls hemolysis in complement inhibitor-naive patients with paroxysmal nocturnal hemoglobinuria. *Blood Adv* **2023**, *7*, 2468–2478. doi:10.1182/bloodadvances.2022009129
5. Hillmen, P.; Szer, J; Weitz, I; Röth, A.; Höchsmann, B.; Panse, J.; Usuki, K.; Griffin, M.; Kiladjian, J.-J.; de Castro, C.; et al. Pegcetacoplan versus eculizumab in paroxysmal nocturnal hemoglobinuria. *N Engl J Med* **2021**, *384*, 1028–1037. doi:10.1056/NEJMoa2029073
6. Debureaux, P.-E.; Kulasekararaj, A.G.; Cacace, F.; Silva, B.G.P.; Calado, R.T., Barone, F.; de Fontbrune F.S.; Prata, P.H; Soret, J.; Sica, M.; et al. Categorizing hematological response to eculizumab in paroxysmal nocturnal hemoglobinuria: a multicenter real-life study. *Bone Marrow Transplant* **2021**, *56*, 2600–2602. doi:10.1038/s41409-021-01372-0
